# Mutation screening of the *SLC26A4* gene in a cohort of 192 Chinese patients with congenital hypothyroidism

**DOI:** 10.1590/2359-3997000000108

**Published:** 2015-01-01

**Authors:** Chunyun Fu, Haiyang Zheng, Shujie Zhang, Yun Chen, Jiasun Su, Jin Wang, Bobo Xie, Xuyun Hu, Xin Fan, Jingsi Luo, Chuan Li, Rongyu Chen, Yiping Shen, Shaoke Chen

**Affiliations:** 1 Department of Genetic Metabolism Children’s Hospital Maternal and Child Health Hospital of Guangxi Zhuang Autonomous Region Nanning People’s Republic of China Department of Genetic Metabolism, Children’s Hospital, Maternal and Child Health Hospital of Guangxi Zhuang Autonomous Region, Nanning, People’s Republic of China; 2 Boston Children’s Hospital Harvard Medical School Boston MA United States Boston Children’s Hospital, Harvard Medical School, Boston, MA, United States

**Keywords:** Congenital hypothyroidism, Pendred syndrome, SLC26A4, gene mutations, China

## Abstract

**Objective:**

Pendred syndrome (PS) is an autosomal recessive disorder characterised by sensorineural hearing loss and thyroid dyshormonogenesis. It is caused by biallelic mutations in the* SLC26A4 *gene encoding for pendrin. Hypothyroidism in PS can be present from birth and therefore diagnosed by neonatal screening. The aim of this study was to examine the* SLC26A4 *mutation spectrum and prevalence among congenital hypothyroidism (CH) patients in the Guangxi Zhuang Autonomous Region of China and to establish how frequently PS causes hearing impairment in our patients with CH.

**Subjects and methods:**

Blood samples were collected from 192 CH patients in Guangxi Zhuang Autonomous Region, China, and genomic DNA was extracted from peripheral blood leukocytes. All exons of the *SLC26A4 *gene together with their exon-intron boundaries were screened by next-generation sequencing. Patients with* SLC26A4 *mutations underwent a complete audiological evaluation including otoscopic examination, audiometry and morphological evaluation of the inner ear.

**Results:**

Next generation sequencing analysis of* SLC26A4 *in 192 CH patients revealed five different heterozygous variations in eight individuals (8/192, 4%). The prevalence of* SLC26A4 *mutations was 4% among studied Chinese CH. Three of the eight were diagnosed as enlargement of the vestibular aqueduct (EVA), no PS were found in our 192 CH patients. The mutations included one novel missense variant p.P469S, as well as four known missense variants, namely p.V233L, p.M147I, p.V609G and p.D661E. Of the eight patients identified with* SLC26A4 *variations in our study, seven patients showed normal size/location of thyroid gland, and one patients showed a decreased size one.

**Conclusions:**

The prevalence of* SLC26A4 *pathogenic variants was 4% among studied Chinese patients with CH. Our study expanded the* SLC26A4 *mutation spectrum, provided the best estimation of* SLC26A4 *mutation rate for Chinese CH patients and indicated the rarity of PS as a cause of CH.

## INTRODUCTION

Pendred syndrome (PS) is an autosomal recessive disease, characterised by functional impairment of the thyroid gland due to thyroid dyshormonogenesis, sensorineural hearing loss, and developmental malformations of the inner ear (1,2). In about 30% of patients dyshormonogenesis is present at birth and is diagnosed by neonatal screening for congenital hypothyroidism (CH). It is caused by homozygous or compound heterozygous mutations in the *SLC26A4* gene encoding pendrin, a multifunctional anion exchanger that is highly expressed in the thyroid, the inner ear and the kidneys (3-5).

In the thyroid, pendrin is expressed at the apical surface of thyrocytes. It acts as a chloride-iodide exchanger transporting iodide from the cell to the colloid in the follicular lumen, where iodide is organified (6-8). Defect in SLC26A4 cause loss of pendrin function, which results in defective iodide organification that induces thyroid overgrowth and goiter in most affected individuals (9-11). However, the phenotypes are variable among different individuals. Up to now, the mutational spectrum of the *SLC26A4* and the genotype-phenotype relationships has not yet been fully established, no study has been designed to assess its prevalence among Chinese CH patients, and very little is known about the proportion of patients with PS among children with CH. Our study therefore aimed to ascertain patients carrying mutations in the *SLC26A4 *gene among subjects with CH. We conducted this study with two main goals: (1) to examine the *SLC26A4* mutation spectrum and prevalence among patients with CH in Guangxi Zhuang Autonomous Region, and (2) to assess the incidence of PS in a cohort of subjects with CH.

## SUBJECTS AND METHODS

### Subjects

We enrolled 192 CH patients (101 females and 91 males), who were identified through newborn screening among 623,000 newborns in the Guangxi Zhuang Autonomous Region, China, from October 2010 to June 2014. Newborn screening was done with filter paper for CH between 72h and 7 days after birth, blood samples were collected from the heel and TSH level was measured by time-resolved fluorescence assay. Subjects with increased TSH (TSH ≥ 8 mIU/l) levels observed during neonatal screening were recalled for further evaluation. Serum TSH and FT4 were determined by electrochemiluminescence assay. Diagnosis of CH is based on elevated TSH levels (TSH ≥ 10 mIU/l) and decreased FT4 levels (FT4 < 12 pmol/l). Thyroid ultrasonography was performed during the neonatal period before treatment. The glands outside reference ranges of newborns in our population (right lobe: 1.44 ± 0.22 cm in length , 0.72 ± 0.11 cm in width and 0.63 ± 0.09 cm in thickness; left: 1.44 ± 0.21 cm in length , 0.70 ± 0.10 cm in width and 0.63 ± 0.09 cm in thickness) were regarded as decreased or enlarged (12). The study was approved by the local Medical Ethics Committee. Informed consent was obtained from the parents of the patients.

### Mutation detection and audiological evaluation

Peripheral venous blood samples were collected from the patients. Genomic DNA was extracted from peripheral blood leukocytes using QIAamp DNA Blood Mini Kit (Qiagen, Germany) according to the manufacturer’s protocol. All exons of *SLC26A4* with their flanking intronic regions were amplified in a 50 μL reaction mixture containing 100-250 ng of genomic DNA, 1× Taq Buffer with 50 mM of KCl, 2.5 mM of MgCl, 200 μM of dNTP, 1 unit of Taq DNA polymerase (Fermentas, USA), and 20 pmol of forward and reverse primers were prepared. Thirty cycles of amplification were carried out with a standard PCR protocol. PCR products were purified using QIA quick PCR purification kit (Qiagen, Germany) following the manufacturer’s instructions. The purified PCR products were sequenced using Illumina MiSeq (Illumina, USA). The Illumina Amplicon Viewer was used for data analysis, and the SnpEff was used for variant annotation. Patients with *SLC26A4 *mutations underwent a complete audiological evaluation including otoscopic examination and audiometry. Morphological evaluation of the inner ear was investigated by high-resolution CT of temporal bones in coronal and axial planes. Enlargement of the vestibular aqueduct (EVA) was defined as a vestibular aqueduct diameter exceeding 1.5 mm at the midpoint between the posterior cranial fossa and the vestibule of the inner ear.

## RESULTS

**Figure 1:**
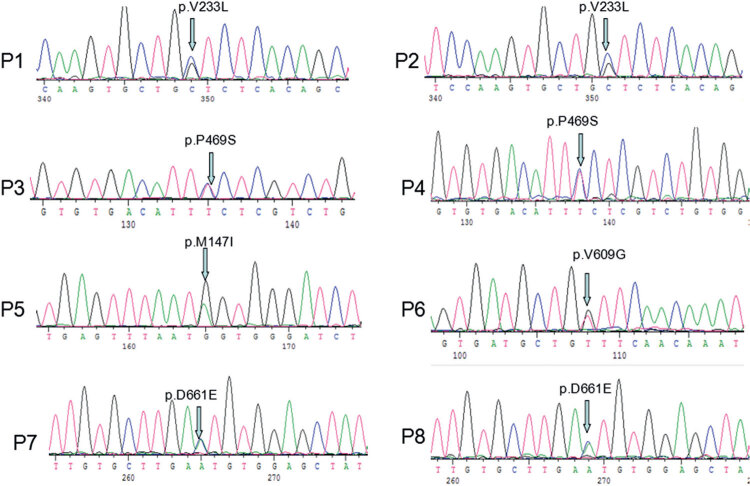
Mutations in SLC26A4 in patients with CH. Figures (P1-P8) showed different sequences in identified eight affected individuals. P1, P2 showed the same SLC26A4 heterozygous mutation p.V233L. P3, P4 showed the same SLC26A4 heterozygous mutation p.P469S. P5, P6 showed heterozygous mutations p.M147I and p.V609G, respectively. P7, P8 showed the same SLC26A4 heterozygous mutation p.D661E.

Next generation sequencing analysis of *SLC26A4* in 192 CH patients revealed five different heterozygous variations in eight individuals (8/192, 4%). The prevalence of *SLC26A4* variants was 4% among studied Chinese patients with CH. The variants included one novel missense variant p.P469S, as well as four known missense mutations, namely p.V233L, p.M147I, p.V609G and p.D661E. All variants were confirmed by Sanger sequencing (Figure 1). Polyphen and Mutation taster predicted that the novel variation would have deleterious effects, by damaging the SLC26A4 protein. Additionally, the variant was not seen in normal population in the database of dbSNP, ESP or 1000 genomes. DNAMAN software was then used to carry out multiple sequence alignment of the SLC26A4 family protein from the following species: *Homo sapiens, Mus musculus, Rattus norvegicus, Canis lupus familiaris* and *Sus scrofa*. The p.P469S variation was found to be located in highly conserved region of SLC26A4. Those all suggesting that the amino acid substitution might be pathologic mutation.

The clinical features and laboratory test results are summarized in the Table 1. All patients were born at full-term to unrelated parents and diagnosed with CH by newborn screening. All patients with *SLC26A4* variants underwent a complete audiological evaluation. Three of eight patients were diagnosed as EVA with moderate to severe hearing problems. As for thyroid morphology, seven of the eight patients showed normal size/location of thyroid gland. Ultrasonography showed Patient 7 with decreased size of thyroid gland (right lobe: 1.1 × 0.6 × 0.5 cm; left: 1.1 × 0.6 × 0.5 cm). No PS was diagnosed in our cohort of 192 CH patients.

**Table 1:** Clinical features, laboratory results and SLC26A4 gene mutations in eight patients

Patient	Sex	Age* (year)	Weight* (kg)centile	Length* (cm)centile	TSH* (mIU/l)	FT4* (pmol/l)	Initial dose of L-T4(µg/kg/day)	Thyroid morphology	*SLC26A4* variations	Clinical phenotype
1	Male	NS	3 (10^th^-25^th^)	50 (25^th^-50^th^)	43	10	6.3	Normal	p.V233L(het)	CH + EVA
2	Female	NS	3.2 (25^th^-5^th^)	50 (50^th^-75^th^)	> 100	3.67	11.6	Normal	p.V233L(het)	CH
3	Male	NS	3.8 (75^th^-90^th^)	51 (50^th^-75^th^)	> 100	5.57	8.6	Normal	p.P469S(het)	CH + EVA
4	Male	NS	3.45 (50^th^-75^th^)	50 (25^th^-50^th^)	> 100	2.22	13.5	Normal	p.P469S(het)	CH + EVA
5	Male	NS	3.15 (25^th^-50^th^)	50 (25^th^-50^th^)	> 100	2.41	9.0	Normal	p.M147I(het)	CH
6	Female	NS	2.7 (3^th^-10^th^)	49 (25^th^-50^th^)	> 100	8.5	13.0	Normal	p.V609G(het)	CH
7	Female	NS	3.02 (25^th^-50^th^)	50 (50^th^-75^th^)	> 100	1.13	12.8	Decreased	p.D661E(het)	CH
8	Male	NS	3.1 (25^th^-50^th^)	50 (25^th^-50^th^)	11	10	6.0	Normal	p.D661E(het)	CH

* Age, weight, length, TSH, FT4 at diagnosis.

NS: newborn screening.

## DISCUSSION

CH is a common endocrine disorder with prevalence ranging from 1:2000 to 1:4000 newborns (13,14). Iodine deficiency is still the major cause of neonate hypothyroidism worldwide (15,16), and the prevalence was reported to be 2.09% in Guangxi Zhuang Autonomous Region (17). Apart from iodine deficiency, the sporadic CH cases can be caused by mutations in a variety of genes including *SLC26A4 *(13,18).

In this study, we conducted the largest *SLC26A4* gene mutation screening so far in CH patients. All the patients were initially identified by newborn screening and subsequently confirmed with re-evaluation. The result of our study revealed five different heterozygous variations in eight individuals (8/192, 4%). The prevalence of *SLC26A4 *mutations was 4% among CH patients in Guangxi Zhuang Autonomous Region, China.

PS is an autosomal recessive disorder which is caused by biallelic mutations in the *SLC26A4* gene. The *SLC26A4* mutation leads to the development of a variable clinical spectrum of hearing loss due to inner-ear malformation as an EVA or mondini cochlea associated with goiter and in some cases CH. A combination of three parameters was suggested to define and diagnose PS (11,19): (i) sensorineural deafness with bilateral EVA; (ii) thyroid abnormality comprising goiter and/or hypothyroidism and/or a positive perchlorate discharge test; (iii) biallelic *SLC26A4* mutations. In our study, we found eight patients with CH had monoallelic *SLC26A4 *mutations and three of them had EVA. No PS was diagnosed in our 192 CH patients. Up to now, the pathogenic role of monoallelic *SLC26A4* mutations remains unclear. Although both biallelic and monoallelic* SLC26A4* mutations were associated with EVA, we cannot exclude the possibility that the second allele of the* SLC26A4 *gene was carrying a mutation, which would not have been identified if located in an intronic or regulatory sequence.

In the same way, patients with monoallelic *SLC26A4* mutations have a very low risk for developing a CH. However, we cannot exclude the possibility that the second mutation has not been identified. Or it was caused by other CH associated gene mutations or environmental factors.

PS is characterized by sensorineural deafness and goiter. Goiter was part of the initial description of PS but is not now considered a constant feature (20-22). In countries with a high iodine intake, goiter development and thyroid dysfunction are usually not seen in patients with biallelic mutations in the *SLC26A4* gene (11). In some cases, thyroid hypoplasia can also be found in patients with biallelic *SLC26A4* mutations (23). In this study, thyroid ultrasound showed seven of the eight patients with *SLC26A4* heterozygous variations had normal size/location of thyroid gland, and the other one had decreased thyroid gland.

Up to now, the *SLC26A4* genotype-phenotype relationships has not yet been fully established, the variability of the phenotypes were observed in different mutations of *SLC26A4*, even in the same mutation among members of the same family. In our study, patients 1-2 shared the same heterozygous mutation (p.V233L), but the clinical phenotypes such as TSH levels, hearing test varied greatly. Variable phenotypes are presumed to be caused by 1) other genetic factors such as gene mutations or modifier genes; 2) individual differences or stochastic phenomena, and 3) environmental factors (iodine uptake, nutrition etc.).

In conclusion, we conducted the largest *SLC26A4* mutation screening for a cohort of patients with CH in Guangxi Zhuang Autonomous Region, China. We identified *SLC26A4* heterozygous pathogenic variant in eight of 192 cases (4%). Three of the eight were diagnosed as EVA, no PS were found in our 192 CH patients. our results indicate the rarity of PS as a cause of CH. We reported one novel *SLC26A4* pathogenic variant (p.P469S), our study expanded the *SLC26A4* mutation spectrum and provided the best estimation of *SLC26A4* mutation rate for Chinese CH patients.
